# An Umbrella Insight into the Phytochemistry Features and Biological Activities of Corn Silk: A Narrative Review

**DOI:** 10.3390/molecules29040891

**Published:** 2024-02-18

**Authors:** Yumei Wang, Jialin Mao, Meng Zhang, Lei Liu, Yu Zhu, Meiling Gu, Jinling Zhang, Hongzhou Bu, Yu Sun, Jia Sun, Yukun Ma, Lina Guo, Yan Zheng, Qi Liu

**Affiliations:** 1 The Research Institute of Medicine and Pharmacy, Qiqihar Medical University, Qiqihar 161006, China; yumeiwangqq@163.com (Y.W.); maojialin1026@163.com (J.M.); 18098728279@163.com (M.Z.); yuzhujada23@163.com (Y.Z.); 13969409385@163.com (M.G.); zhangjinling0413@163.com (J.Z.); zsy5811321@126.com (Y.S.); sunjia900722@163.com (J.S.); kuntengchongtian@163.com (Y.M.); 2 School of Pharmacy, Qiqihar Medical University, Qiqihar 161006, China; gln65@126.com; 3 Graduate School, Qiqihar Medical University, Qiqihar 161006, China; liuleiokc@163.com; 4 Chinese Medicine Detection Laboratory, Drugs Control Center of Qiqihar, Qiqihar 161006, China; 15164679819@163.com; 5 Office of Academic Research, Qiqihar Medical University, Qiqihar 161006, China; zhengyan@s.hrbcu.edu.cn

**Keywords:** corn silk, chemical constituents, pharmacological effects

## Abstract

Corn silk (*Zea mays* L.) is the stigma of an annual gramineous plant named corn*,* which is distributed in many regions worldwide and has a long history of medicinal use. In recent years, with the sustainable development of traditional Chinese medicine, studies of corn silk based on modern technologies, such as GC–MS, LC–MS, and other analytical means, have offered more comprehensive analyses. Phytochemistry studies have shown that the main bioactive components in corn silk include flavonoids, polyphenols, phenolic acids, fatty acids, and terpenoids. Pharmacological studies have shown that corn silk extract has various pharmacological effects, such as reducing blood lipids, lowering blood pressure, regulating blood sugar levels, anti-inflammatory effects, and anti-oxidation effects. In this paper, the related research on corn silk from the past few years is summarized to provide a theoretical reference for the further development and utilization of corn silk.

## 1. Introduction

Corn silk is a biological by-product of *Zea mays* L., an important food crop worldwide. In China, corn silk first appeared in the ancient book *Southern Yunnan Materia Medica* in 1476 and then was recorded in *Lingnan Pharmacopoeia* and the *Sichuan Journal of Traditional Chinese Medicine*. In the *Chinese Pharmacopoeia* (1997 edition), corn silk was also recommended as a common Chinese herb. In addition to its official applications, corn silk is also used in diverse foods and healthcare products, indicating that corn silk may have potential value in diversified medicinal and edible products [1]. Due to the extensive production and processing of *Zea mays* L. in China and other countries, corn silk resources have become particularly abundant. In recent years, the sustainable development of traditional Chinese medicine (TCM) has attracted much attention, and the exploitation of corn silk has become a research hotspot.

TCM possesses typical multicomponent and multitarget characteristics in modern research. When looking at its phytochemistry profiles, common micromolecules contain flavonoids, saponins, alkaloids, organic acids, etc., and the macromolecules of TCM usually include polysaccharides and proteins. Thus, one single constituent may play a pivotal role in a pharmaceutical effect, and diversified classes of constituents may work together, resulting in a particular effect. In addition, regarding pharmacological activities, the functions and related mechanisms of TCM are usually manifold. 

In clinical or basic research on TCM, corn silk, a potentially safe herb [2] that induces diuresis and reduces edema, has been widely used in treatments for diabetes, diabetic nephropathy, hyperlipidemia, hyperuricemia, etc. [3]. Its various pharmacological activities may be closely related to its multifarious chemical composition. This suggests that the chemical components have an important position in basic research on related pharmacodynamic substances. Taking this into account, the chemical ingredients and biological activities of corn silk play a vital function when attempting to illustrate its use in the treatment of various diseases. As a result, summarizing the chemical compounds and the pharmacological actions of corn silk is vital.

In recent years, corn silk has been paid much attention on account of the exploitation of medicinal resources. Studies of its phytochemical and pharmacological properties have become hot topics. According to reports in the literature, there are a variety of chemical components in corn silk, covering macromolecular polysaccharides [4] and various micromolecular components [5], like saponins, flavonoids, sterols, amino acids, terpenes, and organic acids. Furthermore, in modern clinics, corn silk is applied to treat various diseases, such as nephritis, acute and chronic pneumonia, diabetes, hypertension, and edema [6]. In addition, many modern pharmacological activities, such as antihypertension activities [7], lowering blood lipids [8], anti-inflammatory activities [9], anti-urolithiasis activities [10], anti-oxidant activities [11], protecting the liver [12], and other effects [13] have been verified in previous studies.

In this paper, the comprehensive chemical compositions and pharmacological effects of corn silk are reviewed based on the relevant literature. Detailed information regarding its chemical profiles and familiar activities is presented. The data collected not only present the current progress of research on corn silk but can also improve the current understanding of corn silk and assist in further research studies. 

## 2. Chemical Classification and Structures

The chemical composition of corn silk is particularly diverse [14,15]. The compounds separated and identified from corn silk can mainly be divided into six types: covered flavonoids, polyphenols, sterols, terpenoids, amino acids, and organic acids.

### 2.1. Flavonoids

**Figure 1:**
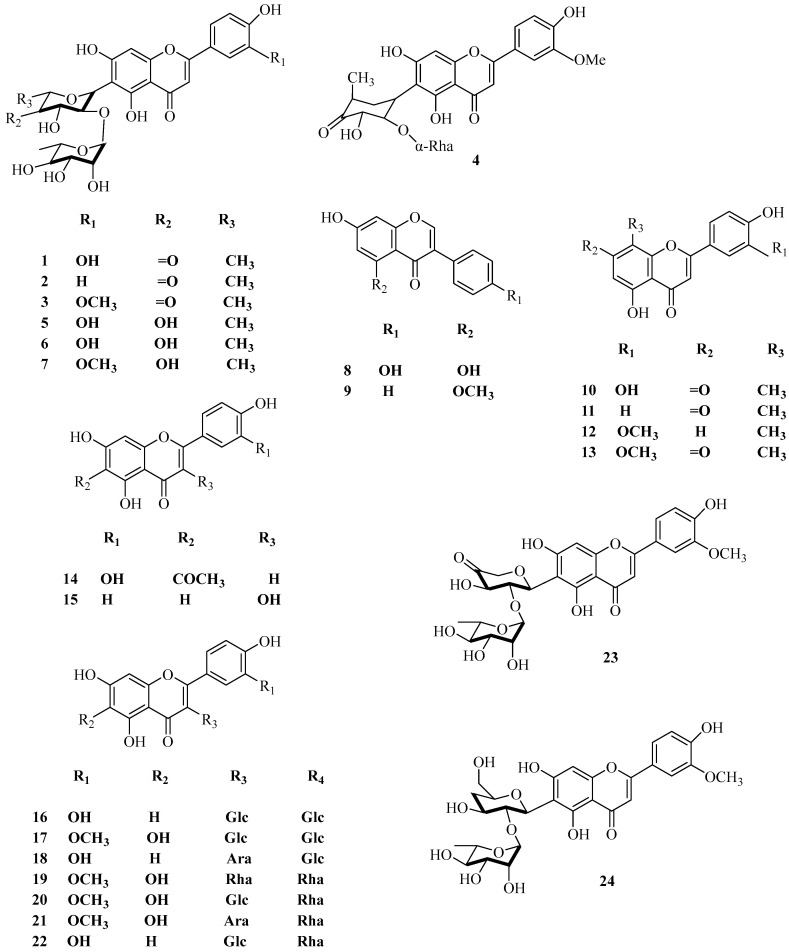

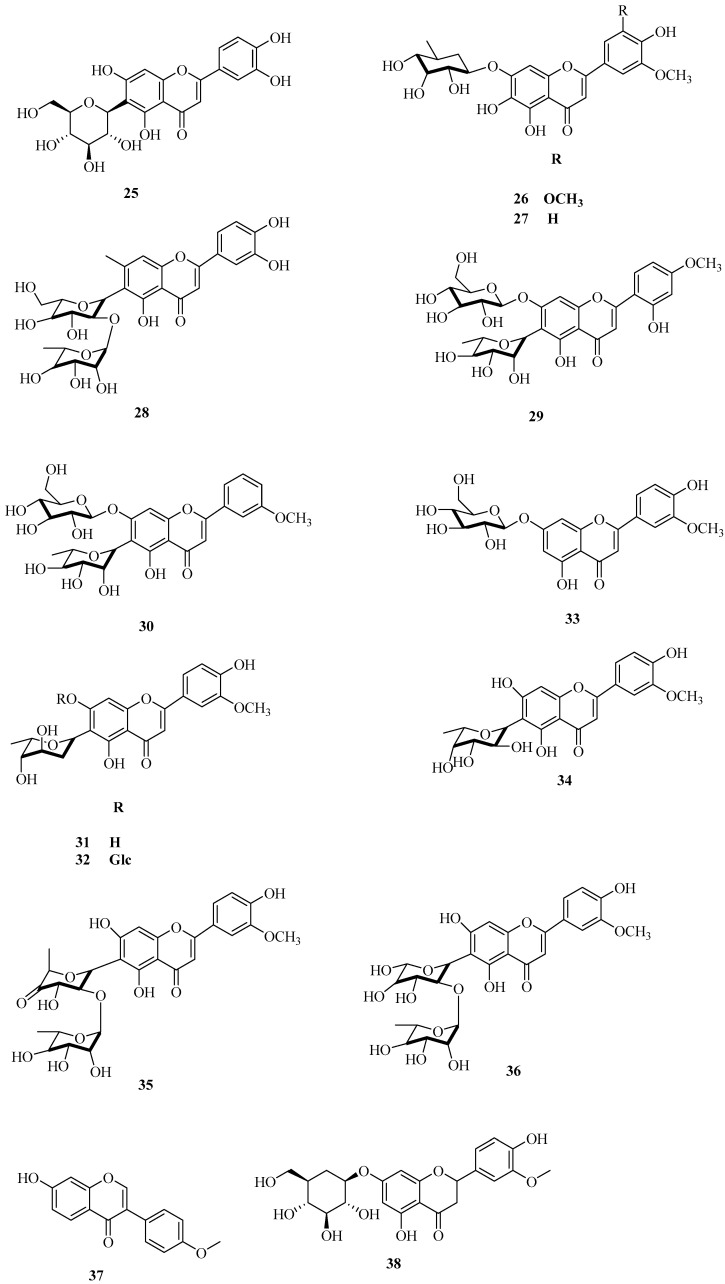

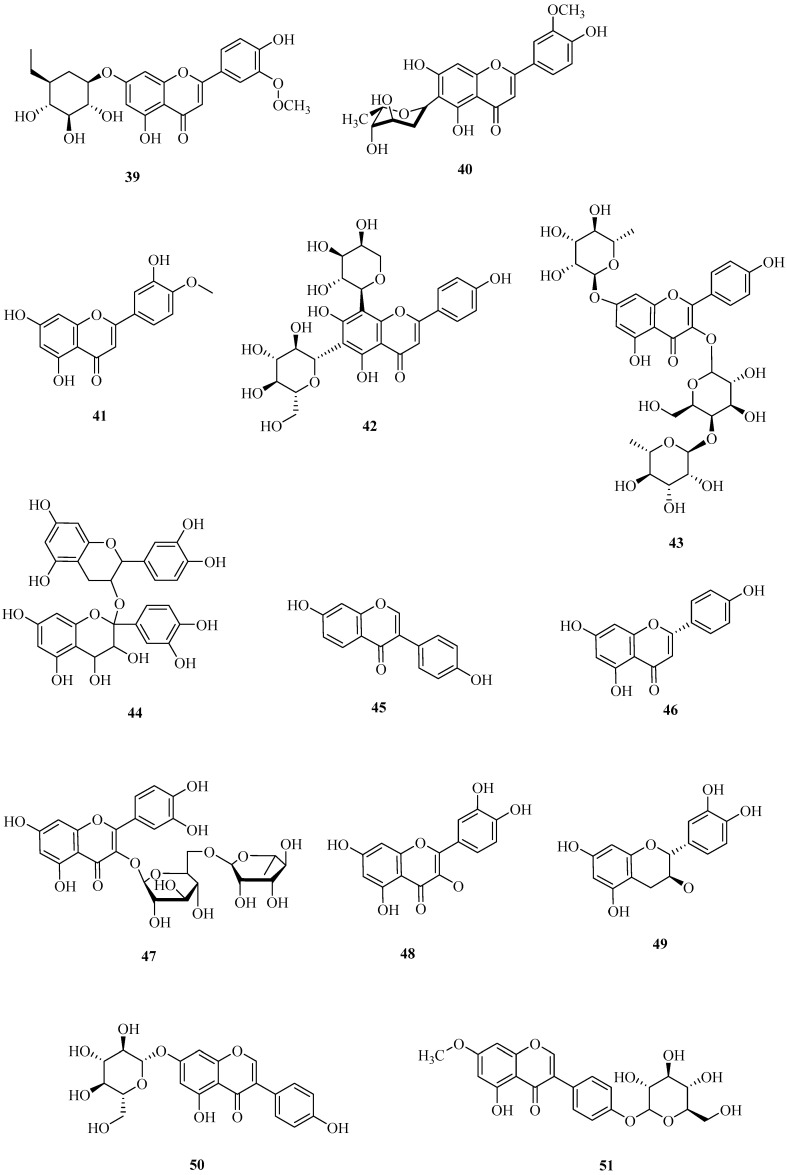

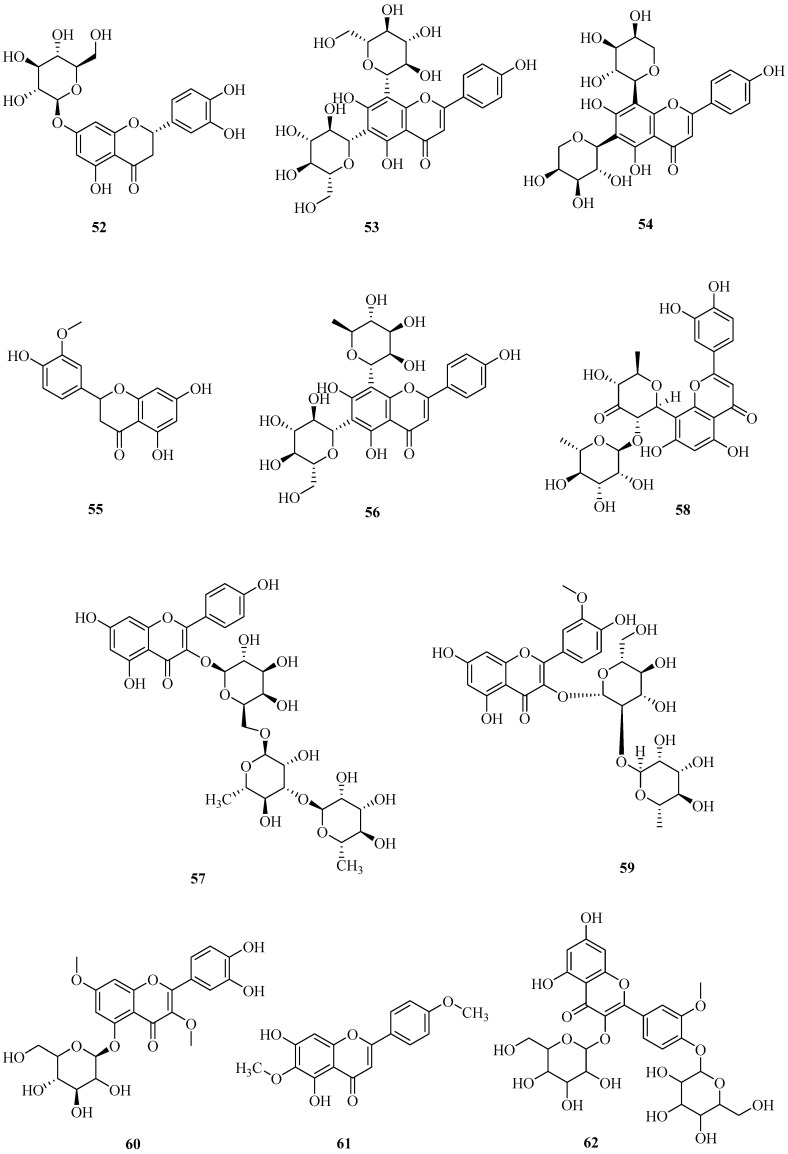

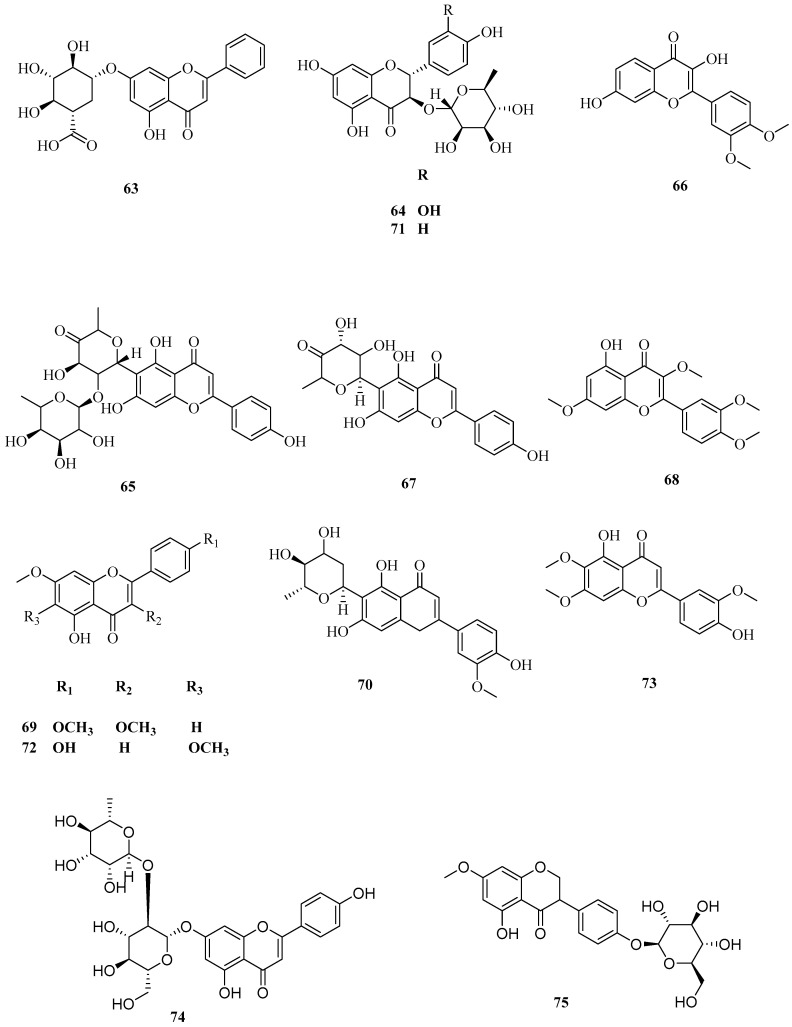

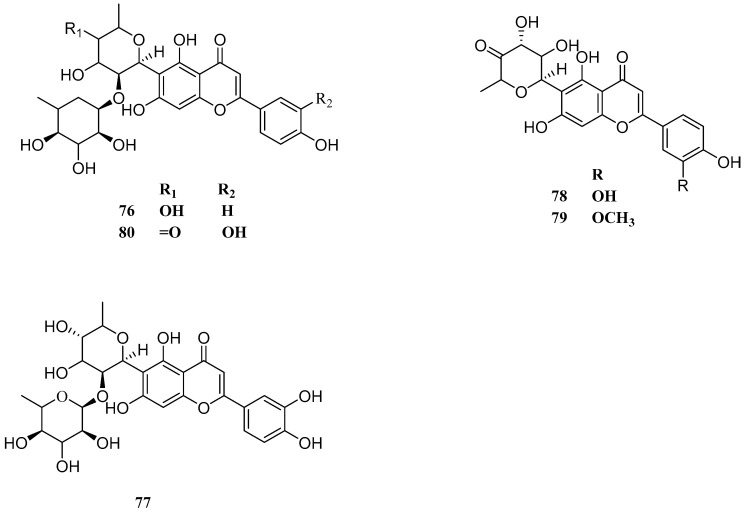
Structures of flavonoid constituents in corn silk.

Flavonoids are the main chemical components of corn silk, containing flavonoid glycosides, flavonols, and isoflavones. Among them, apigenin, luteolin, robinin, and chrysoeriol are the common mother nucleus structures. Our previous study showed that 35 flavonoid constituents were successfully identified in the enrichment of corn silk using the LC–MS/MS approach; luteolin-*C*-glycosides and apigenin-*C*-glycosides account for 40% of the total and play a critical role [16]. Yi Ting et al. [17] used HPLC-Q-TOF-MS technology to analyze the total flavonoids of corn silk prepared by reflux extraction and macroporous resin enrichment; as a result, 19 flavonoids—10 flavonoid glycosides and 9 flavonols were identified. Li Qiang et al. [18] found three new flavonoid glycosides in the ethyl acetate extract of corn silk. The main sugar chain binding sites of flavonoid glycosides are 3-C and 6-C sites and a few are 7-C and 8-C sites. A few flavonoid glycosides are oxygenosides with a binding position of 2-C. The main sugar molecules of the flavonoid glycosides contain glucose, rhamnose, etc. [19]. According to the phytochemical literature on corn silk published in recent years, a total of 80 flavonoid constituents have been reported, including luteolin, apigenin, maysin, and multiple O-glycosides and C-glucosides of flavonoids. Accurate CAS numbers and related structural formulas are displayed in Table 1. The chemical structures of the flavonoids obtained from corn silk are shown in Figure 1.

### 2.2. Sterols, Terpenoids and Saponins

**Figure 2:**
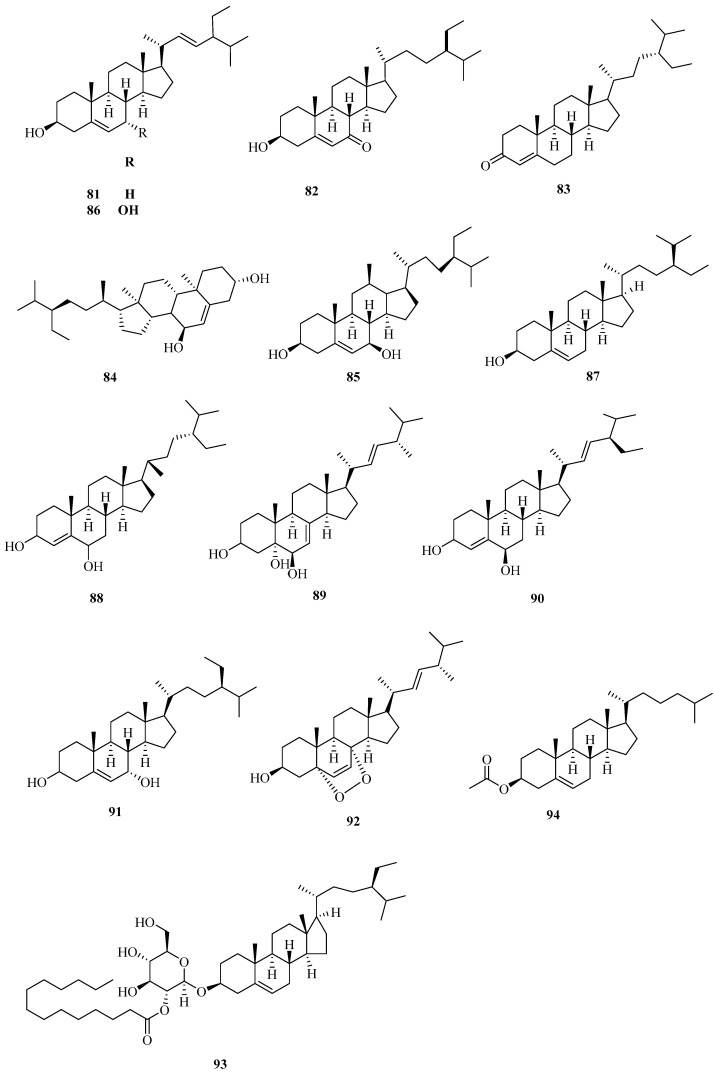
Structures of sterol constituents in corn silk.

Sterols are the active natural substances come from plants and animinals, which own essential physiological functions widely used in medicine, health care, food, and other fields. Corn silk studies of β-sitosterol are plentiful, and the content of it is high. Zhang Haibo et al. [40] used the HPLC-ELSD tool to determine the contents of β-sitosterol in corn silk at different stages in Henan province in China, and the results showed that β-sitosterol was at the highest level in the middle of July. Moreover, Jingge Tian [41] studied the liposoluble constituents from the extract layers of petroleum ether and ethyl acetate of corn silk. Consequently, 17 compounds were isolated. Among them, four ingredients—stigmast-4-ene-3β,6β-diol, stigmast-4,22-diene-3β,6β-diol, stigmast-5-ene-3β,7α-diol, and ergosterol endoperoxide—were sterol compounds. In summary, a total of 14 sterol constituents in corn silk have been found to date. The sterols in corn silk are listed in Table 2, and the structures are displayed in Figure 2.

**Figure 3:**
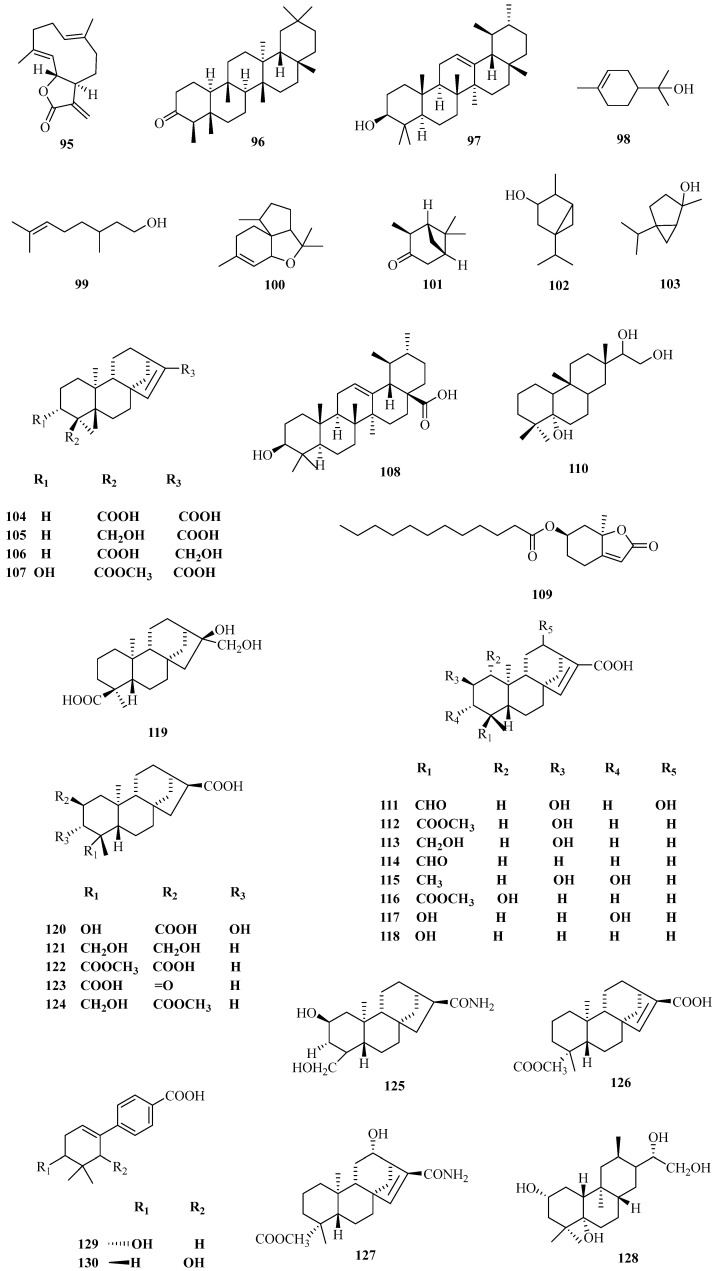

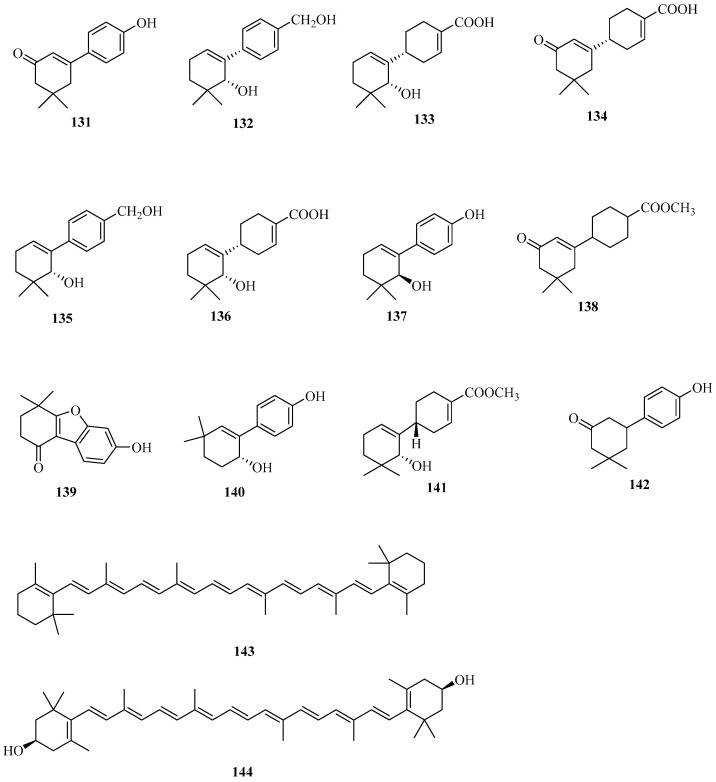
Structures of terpenoid constituents in corn silk.

Studies on terpenoids from corn silk are relatively rare. However, the contents of terpenoids are comparatively great. With the continuous advancement of the utilization of TCM resources, research into terpenoids from corn silk is gradually increasing, and many new sesquiterpenes and diterpenoids, as well as some monoterpenes and triterpenoids, have been isolated and identified [44]. Zhao Min et al [42] purified three terpene profiles, namely 19-hydroxy-*R*-kaurane-15-ene-17-carboxylic acid, 17-hydroxy-*R*-kaurene-15-ene-19-oleic acid, and 3α-hydroxy-*R*-kaurene-15-ene-17-oleic acid-19-methyl carboxylate from corn silk using silica gel combined with Sephadex LH-20 column chromatography. Detailed chemical information and the corresponding structures are displayed in Table 3 and Figure 3.

**Figure 4:**
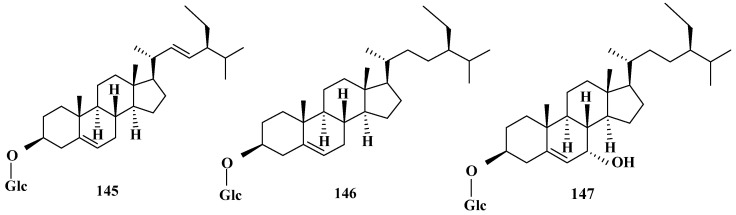
Structures of saponin constituents in corn silk.

The saponin ingredients derived from corn silk are rarely reported. Up to now, three saponins named 7α-hydroxysitosterol-3-*O*-β-d-glucopyranoside, stigmasterol-3-*O*-β-d-glucopyranoside, and 3-β-sitosterol-d-glucopyranoside have been reported. Moreover, 7α-hydroxysitosterol-3-*O*-β-d-glucopyranoside could be isolated in the ethyl acetate extract of corn silk by silica gel column chromatography [34], stigmasterol-3-*O*-β-d-glucopyranoside and 3-β-sitosterol-d-glucopyranoside could be separated in the petroleum ether extract from corn silk by gel column chromatography [45]. Upon further investigation, we have found that the aforementioned 3 saponin constituents are mainly glycosides at the 3-C position, and the bounding sugar molecule is β-d-glucose. The attentive chemical compositions and associated structures of saponins from corn silk are shown in Table 4 and Figure 4.

### 2.3. Organic Acids

**Figure 5:**
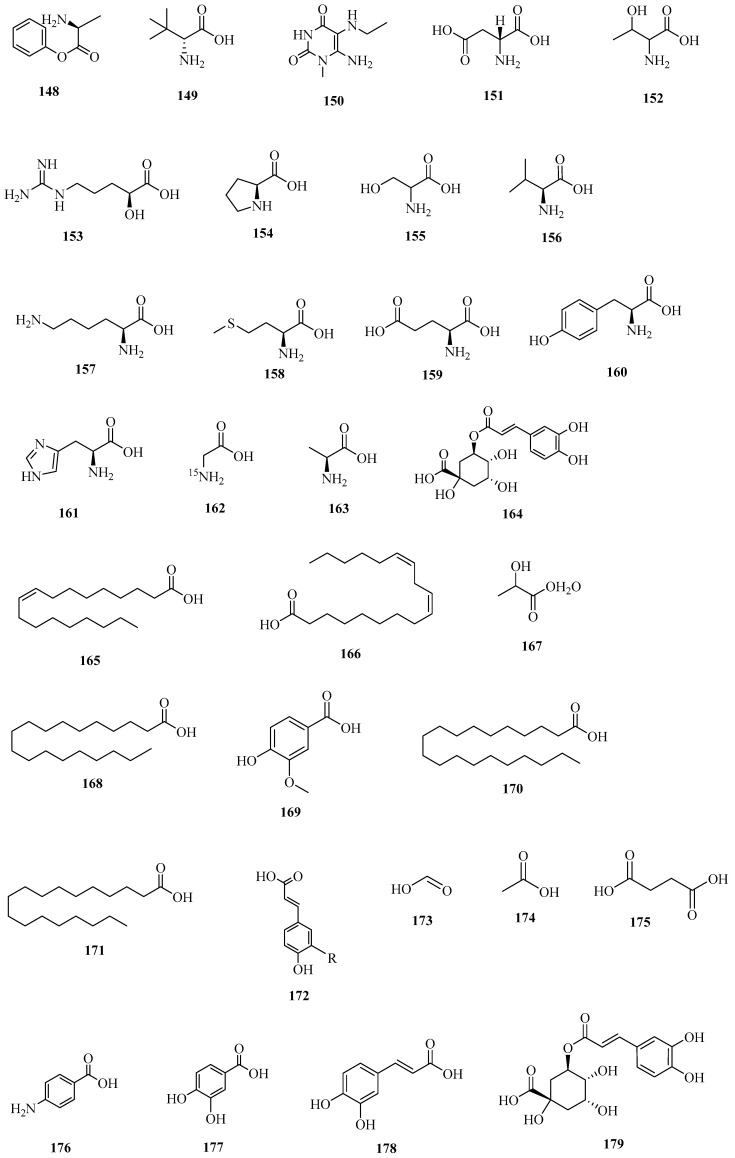

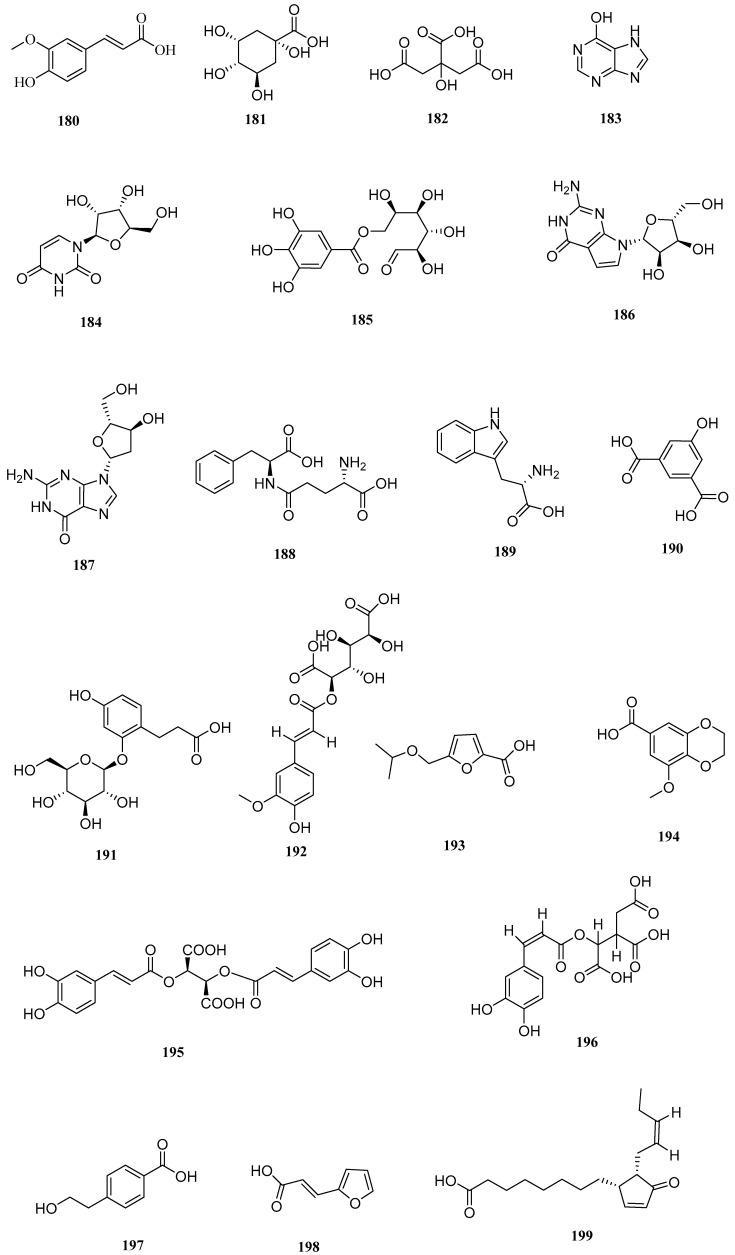

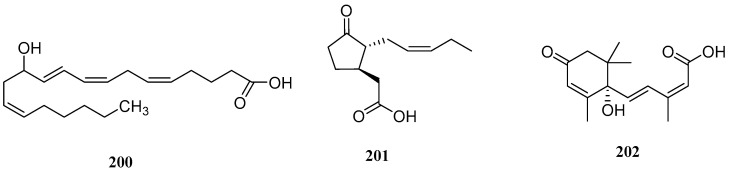
Structures of organic acid constituents in corn silk.

The organic acids in corn silk are divided into amino acids, short-chain organic acids, and long-chain organic acids. The results show that there are 16 organic acids in corn silk. Among them, the contents of glutamic acid and aspartic acid are the highest, and four essential amino acids, namely leucine, phenylalanine, threonine, and valine, take second place [54]. As a result, a total of 55 organic acids in corn silk were discovered, including linoleic acid, lactic acid, docosanoic acid, vanillic acid, stearic acid, etc. The formulas and CAS numbers are shown in Table 5, and the structures are shown in Figure 5.

### 2.4. Polysaccharides and Other Ingredients

**Figure 6:**
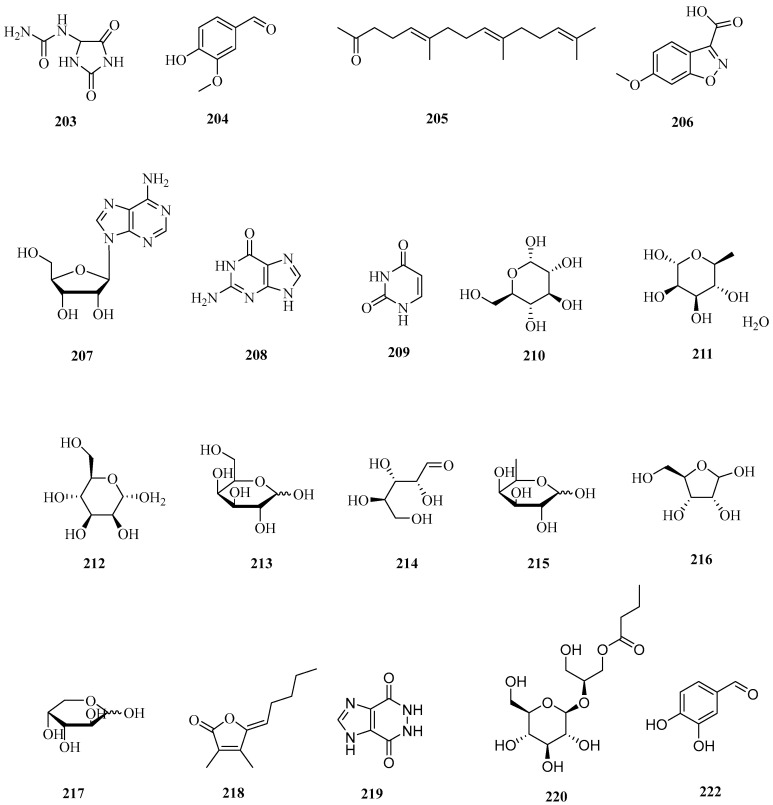

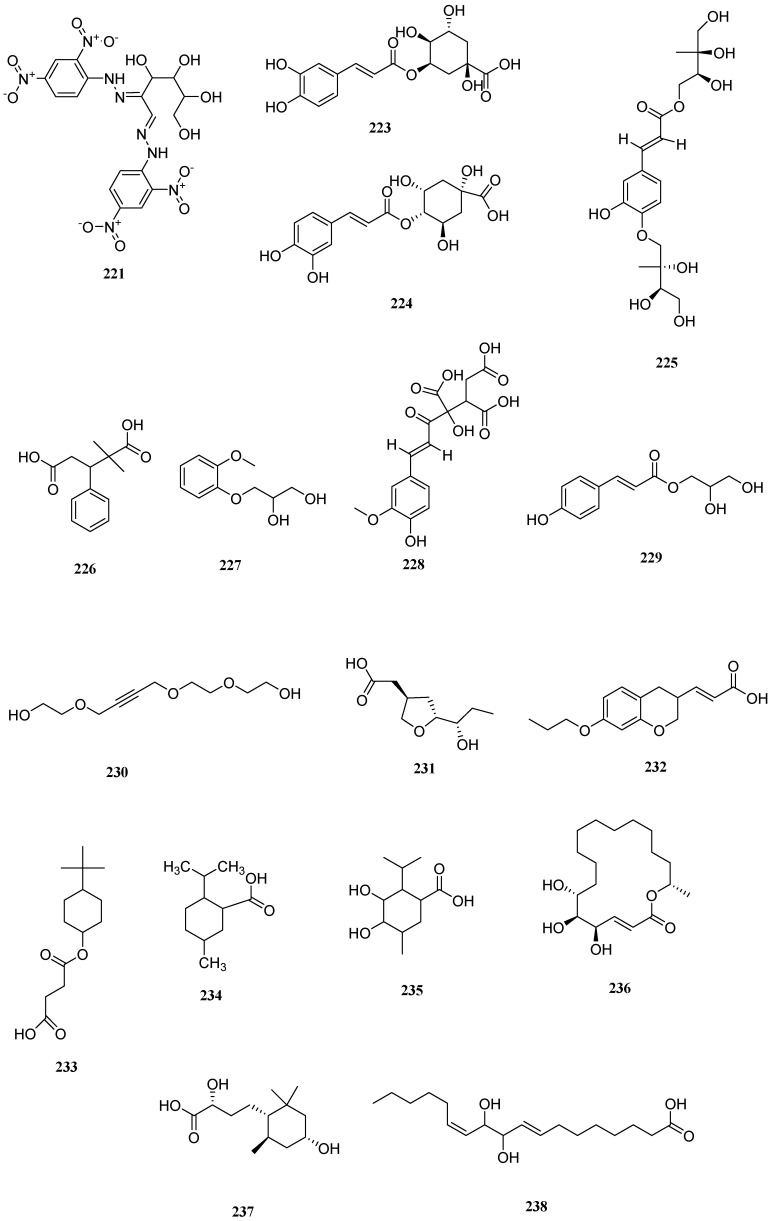

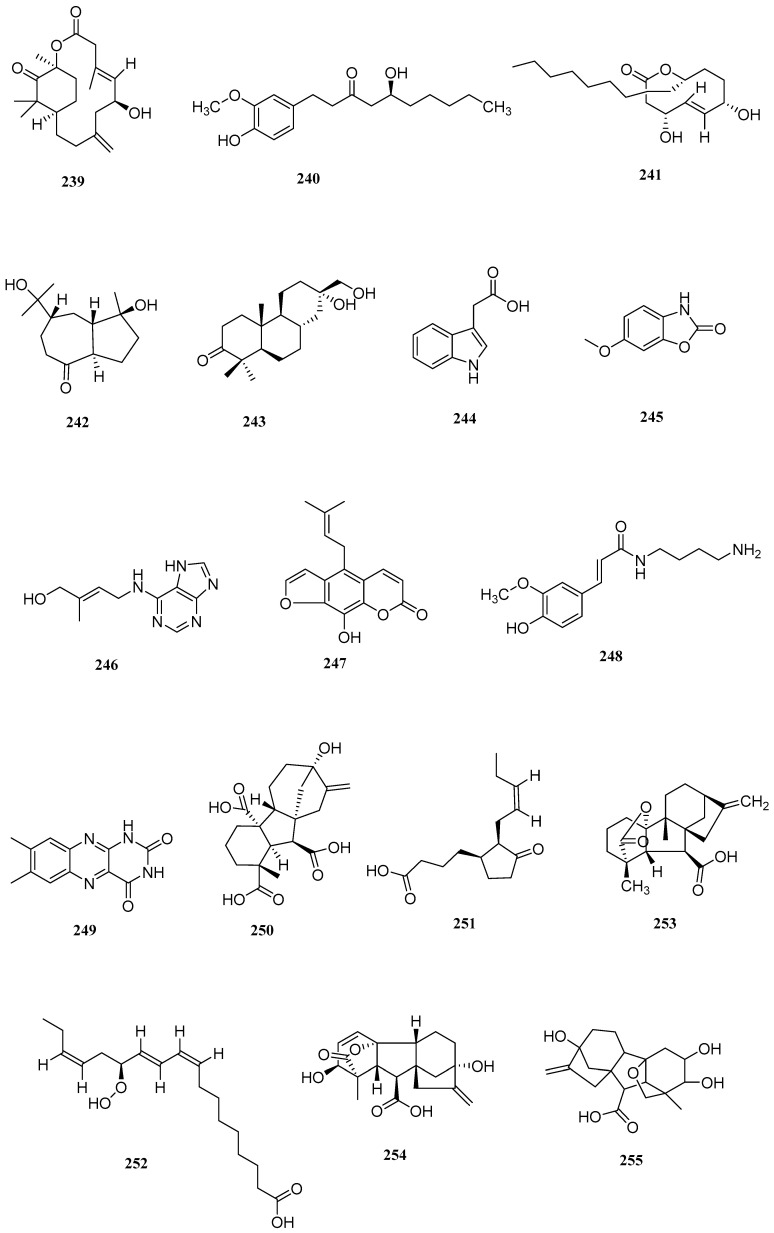

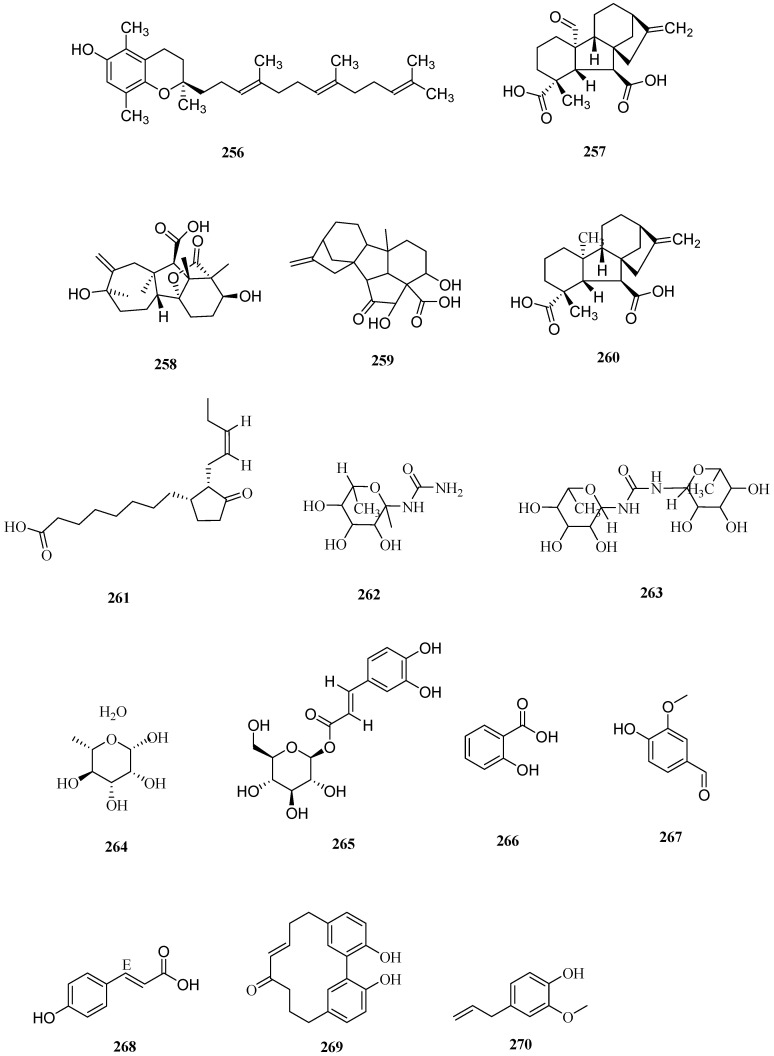
Structures of polysaccharides and other constituents in corn silk.

Polysaccharides are a kind of polymer carbohydrate composed of multiple monosaccharides with small molecules. Polysaccharides of corn silk are mainly found in aqueous extract. The content of polysaccharides in corn silk is high, reaching up to 4.87% in dry products [41]. In recent years, polysaccharides from corn silk have attracted much attention. Summarizing previous studies, it was found that the main compositions of polysaccharides from corn silk include mannose, lactose, galactose., rhamnose, arabinose, xylose, and glucose [62]. In addition to polysaccharides, other chemical components in corn silk have been reported. Xu Yan et al. found two urea glycosides—rhamnoside and 1,3-2-rhamnoside ureaside in corn silk [27]. Summing up the recent literature, a total of 82 chemical profiles covering polysaccharides and other ingredients have been discovered, which are displayed in Table 6. The structures of 68 ingredients, except for the compounds with serial numbers **271**–**284**, are displayed in Figure 6. 

## 3. Pharmacological Actions

The pharmacological effects of corn silk are abundant, including anti-oxidant, anti-inflammatory, anti-tumor, hypoglycemic, and hypolipidemic properties, among others. In daily life, corn silk is widely used for the improvement of cardiovascular diseases, diabetes, Alzheimer’s, hyperuricemia, chronic nephritis, and other diseases. With the development of modern analytical techniques, the pharmacological research of corn silk has gradually deepened.

### 3.1. Hyperglycemic Effect

Diabetes mellitus, a metabolic disease characterized by hyperglycemia, is caused by multiple factors, such as the reduction or low response of insulin. Among the different causes of diabetes mellitus, chronic high blood sugar is the crucial reason. To alleviate symptoms or delay the onset of complications from diabetes mellitus, hyperglycemia is of great concern. Pharmacological studies have shown that polysaccharides in the aqueous extract of corn silk are the main active components confronting hypoglycemia [6]. Jin et al. [65] found that the aqueous extract of corn silk significantly reduced fasting blood glucose levels, improved glucose tolerance, and reduced insulin resistance in type 2 diabetes mice. In addition, corn silk polysaccharides exhibited a good effect on the suppression and prevention of acute hyperglycemia in alloxan-induced diabetes, whether in type 1 or type 2-model mice [66]. N-butanol fraction from corn silk can alleviate the decreasing trend of body weight, reduce blood glucose and serum insulin levels, improve glucose tolerance, regulate lipid levels, and increase the activity of anti-oxidant enzymes in type 2 diabetic mice. Moreover, except for the effect in vivo, the *N*-butanol fraction from corn silk also exhibited favorable action on cells in vitro [67]. In addition to the traditional extract of corn silk, the products of fermentation and decoction from corn silk can be made from saccharomyces cerevisiae, bacillus subtilis, and lactobacillus. After the hypoglycemic experiment, the lactobacillus fermentation product exhibited a much more effective effect on type 2 diabetic mice [68]. Moreover, the flavonoid extract of corn silk can also affect blood glucose, prevent lipid metabolism disorders and abnormal changes in blood rheological indexes caused by a high-fat diet, reduce the fasting blood glucose concentration and HDL-c concentration in diabetic rats, significantly reduce the content of serum and liver malondialdehyde, and observably improve SOD activity [69]. Therefore, it can be seen that the hyperglycemic effect of corn silk is meaningful for the treatment of diabetes mellitus.

### 3.2. Antigout Action

Gout, which belongs to the category of metabolic rheumatism, is a recurrent metabolic arthritis disease caused by an increase in purine bioanabolism. The excessive production or poor excretion of uric acid leads to the increase of uric acid in the blood, inducing the deposition of urate crystals in joint synovium, bursa, cartilage, and other tissues. The pathogenesis of gout is directly related to increased purine synthesis or high levels of uric acid in the blood. Recently, scholars have conducted a series of studies on the pharmacological effects of corn silk against gout [70]. Li Ping et al [71] showed that corn silk flavonoid extract at doses of 0.25 g/100 g, 0.5 g/100 g, and 1.0 g/100 g reduces the serum levels of interleukin-1β and uric acid in rats, indicating that corn silk can treat the joint swelling found in acutely gouty arthritis rats. Lv Guangfu et al. [72] found that the total flavonoid extract of corn silk at doses of 0.5 mg/kg, 1.0 mg/kg, and 2.0 mg/kg promotes the excretion of uric acid in the kidneys of isolated rats and improves the renal function parameters of the kidney tissue, explaining that corn silk extract showed a significant intervention effect on n-acetyl-β-d-glucogluconic anhydrase in acute injury and active lesions. Lin Zhe et al. [73] studied the mechanism of the flavonoid extract from corn silk against gout nephropathy. The results showed that flavonoids of corn silk reduce the content of β2-MG, RBP, ALB, TRF, and NAG in blood and promote the renal uric acid excretion rate to improve the glomerular filtration rate and relieve the damage to the renal tubular system, therefore playing a good role in the treatment of uric acid nephropathy.

### 3.3. Liver Protection

The liver is an essential metabolic organ of the human body. Usually, unhealthy lifestyle habits, such as high-fat and high-sugar diets, excessive drinking, etc., may lead to a certain degree of damage to the liver. Corn silk extract can significantly improve intrahepatic cholestatic liver disease and effectively inhibit the development of liver fibrosis [74]. Jin Danli et al. [65] found that the total flavonoids of corn silk at doses of 300 mg/kg and 600 mg/kg protect the liver and reduce fat vacuoles in the liver. Jingyi’s results showed that the total flavonoids at doses of 50 mg/kg and 100 mg/kg of corn silk have a protective effect on carbon tetrachloride-induced chronic liver injury in rats, which can significantly reduce levels of AST, ALT, and HA in the serum of rats with chronic liver injury, lessen the content of MDA in serum and liver, and synchronously improve the activity of SOD [75].

### 3.4. Anti-Hyperlipidemia Action

Hyperlipidemia, divided into primary and secondary hyperlipidemia [76], is a common cardiovascular disease in clinical. Primary hyperlipidemia is mainly related to congenital and hereditary reasons and is mainly caused by single or polygene gene defects, which result in the abnormal action of receptors, enzymes, or apolipoproteins involved in the transport and metabolism of lipoproteins. Secondary hyperlipidemias are mostly connected with metabolic disorders such as diabetes, hypertension, hypothyroidism, obesity, liver/kidney disease, and hyperadrenal function. Moreover, there are other factors of hyperlipidemia, including age, sex, season, alcohol, smoking, diet, etc. Reports have shown that, following the long-term intake of high-fat and high-calorie foods, a large amount of local blood lipids gather, and finally, blood lipid metabolism becomes disordered [77]. As for the polysaccharides of corn silk, smaller molecules of polysaccharides may show a better effect on hypolipidemia. Moreover, like the polysaccharides of corn silk, the flavonoids of corn silk exhibit a specific hypolipidemic function [78]. Zhang Yan et al. showed that the flavonoid extract of corn silk at doses of 300 mg/kg and 500 mg/kg reduces serum lipid levels such as TC, TG, and LDL-C. Inversely, the HDL-C level increased [79]. Corn silk concentration with doses of 0.25 g/mL, 0.5 g/mL, and 1.0 g/mL could effectively alleviate the increase of serum triglycerides and total cholesterol in rats induced by a high-fat diet, showing a specific inhibitory effect on hyperlipidemia [80].

### 3.5. Anti-Oxidant Activity

The superfluous production of free radicals is associated with plenty of diseases like cancer and aging. Anti-oxidants aim to effectively inhibit the oxidation of free radicals. The mechanisms include acting on free radicals directly or consuming free radicals indirectly to prevent further reactions. Anti-oxidant activity is one of the critical pharmacological effects of corn silk, and the effective components are polysaccharides, phenolic acids, and flavonoids [79,81]. Ahmed El-Ghorab et al. [82] revealed good anti-oxidant actions of dichloromethane extraction, petroleum ether extraction, ethanol extraction, and water extraction from corn silk. In addition, the flavonoid glycosides of corn silk exhibited obvious anti-oxidant and free-radical scavenging activities [83]. Zhang’s experiments showed that various flavonoids in corn silk extract had good anti-oxidant activity [39]. Maksimović studied the anti-oxidant activity of corn silk polyphenols. The data showed that the anti-oxidant activity is positively correlated with the total phenolic content in corn silk, indicating that the higher the total phenol content in corn silk, the better the anti-oxidant activity [84].

### 3.6. Anti-Inflammatory Effect

Inflammation is an immune defense response of the body against harmful stimuli, which is typically characterized by redness, swelling, heat, pain, and dysfunction [85]. Tian Ze et al. verified the anti-inflammatory effect of corn silk by animal experiments. The outcomes exhibited that the active ingredient named luteolin in corn silk could attenuate the inflammation [86].

### 3.7. Kidney Protection

Corn silk is usually applied in TCM’s clinical application in treating kidney diseases. The mechanism of kidney injury may be that the uric acid deposited in the kidney upregulates the NF-κB signaling pathway, leading to the release of inflammatory factors and kidney damage [87]. Network pharmacological analysis shows that the flavonoids in corn silk alleviate kidney damage by releasing large amounts of inflammatory factors. Moreover, luteolin and other flavonoids have a significant effect on common targets like HIF, AKT, and PHD, therefore regulating the signal transduction pathways such as HIF-1, PI3K-AKT, TNF, IL-17, etc., such that they play a therapeutic role in chronic glomerulonephritis [88].

### 3.8. Antihypertensive Activity

Hypertension is a clinical syndrome characterized by increased systolic or diastolic blood pressure in systemic arteries, which may cause some functional or organ damage to the heart, brain, kidney, and other organs. In middle-aged and elderly people, hypertension is a common chronic disease, which increases the prevalence of cardiovascular and cerebrovascular diseases. Therefore, searching for effective and safe drugs is a research hotspot. In recent years, with the development of corn silk, antihypertensive activity has caught researchers’ attention [5,89]. The aqueous extract of corn silk at doses of 60 mg/kg, 130 mg/kg, 192.5 mg/kg, and 260 mg/kg showed a specific dose-dependent antihypertensive effect [90]. Li et al. [91] determined an active plant peptide of the aqueous extract from corn silk using the proteomics method, and verified its inhibiting action of angiotensinase and the relaxing reaction of blood vessels.

### 3.9. Other Activities

Chinese medicine often has multiple pharmacological effects. Except for the above-mentioned activities, corn silk also shows other activities like anti-Alzheimer’s and anti-cancer properties, protecting the reproductive system, immunomodulation, and antibacterial properties. The anti-Alzheimer’s disease effect of corn silk is mainly reflected in the activity of phosphotransferase and the response to hormones [86]. Moreover, when talking about anti-cancer activity, compounds from corn silk may target the responses of immune cells, induce cytotoxicity, and upregulate the expression of pro-apoptotic genes p53, p21, caspase 9, and caspase 3 in certain cells like HeLa cervical cancer cells, MCF-7 breast cancer cells, PANC-02 pancreatic cancer cells, and Caco-2 colon cancer cells [92]. Moreover, corn silk extract could recover the amounts of sex hormones and sperm to normal conditions by reducing lipid peroxidation in male mice [93]. In addition, corn silk also inhibits Escherichia coli, Staphylococcus aureus, and Candida albicans and exhibits an antimicrobial effect [94]. The pharmacological actions and related mechanisms of corn silk are detailed in Table 7.

## 4. Conclusions

With the sustainable development of TCM, the chemical compositions and pharmacological effects of corn silk have gradually become a research hotspot. This paper presents the chemical profiles and pharmacological actions of corn silk. The compounds mainly include flavonoids, terpenoids, organic acids, etc., and a total of 284 chemical components of corn silk are detailed and expounded. The research on pharmacological effects is mainly focused on anti-inflammatory properties, anti-oxidant properties, liver protection, and the alleviation of acute and chronic nephritis. In addition to the traditional pharmacological effects of corn silk, other functions, such as its anti-AD and anti-cancer properties and its protection of the reproductive system, etc., are reported. Except for chemical unscrambling, the pharmacological effects and related mechanisms were also overviewed. Summing up the above, the research on the pharmacological effects of corn silk in recent years has mainly focused on aqueous/alcohol extracts, covering flavonoids and polysaccharides. The actions and mechanisms of other extracts of corn silk should be studied in depth using modern analytical techniques and methods. However, up to now, there have been few studies on the monomeric active compounds derived from corn silk. Based on the chemical components and related activities, several classic chemical ingredients of corn silk, like maysin, luteolin, apigenin, and their various derivates, may be marker compounds. Hence, the further study of active ingredients from corn silk could be a meaningful direction in the future.

## Figures and Tables

**Table 1:** Chemical composition of flavonoids in corn silk.

No.	Name	CAS	Formula	Reference
**1**	maysin	70255-49-1	C_27_H_28_O_14_	[20]
**2**	apimaysin	74158-04-6	C_23_H_46_N_4_O_2_	[20]
**3**	3′-methaxymaysin	101920255	C_28_H_30_O_14_	[20]
**4**	ax-5″-methane-3′-methoxymaysin	74977694	C_28_H_32_O_14_	[21]
**5**	2″-*O*-α-l-rhamnose-6-*C*-quinose-Luteolin	— —	C_28_H_34_O_14_	[21]
**6**	2″-*O*-α-l-rhamnose-6-*C*-fucose-Luteolin	— —	C_28_H_34_O_14_	[21]
**7**	2″-*O*-α-l-rhamnoside-6-*C*-fucoside-3′-methoxyluteolin	— —	C_29_H_36_O_14_	[22]
**8**	genistein	446-72-0	C_15_H_10_O_5_	[23]
**9**	7-hydroxy-4′-methoxyflavone	487-17-2	C_16_H_12_O_4_	[22]
**10**	apigenin	520-36-5	C_15_H_10_O_5_	[24]
**11**	luteolin	491-70-3	C_15_H_10_O_6_	[25]
**12**	chrysoeriol	491-71-4	C_16_H_12_O_6_	[23]
**13**	5,8,4′-trihydroxy-7-methoxyflavanone	— —	C_17_H_13_O_6_	[26]
**14**	6-acetyl-luteolin	122377901	C_29_H_32_O_16_	[27]
**15**	isorhamnetin	480-19-3	C_16_H_12_O_7_	[28]
**16**	5,7,4′-trihydroxyflavone-3,6-*C*-diglucoside	— —	C_27_H_33_O_18_	[29]
**17**	5,7,3′-trihydroxy-4′-methoxyflavanone-3,6-*C*-diglucoside	— —	C_18_H_35_O_19_	[29]
**18**	5,7,4′-trihydroxyflavone-3-*C*-Arabinose, 6-*C*-glucoside	— —	C27H31O17	[30]
**19**	5,7,3′-trihydroxy-4′-methoxyflavanone-3,6-*C*-dirhamnoside	— —	C_28_H_35_O_17_	[30]
**20**	5,7,3′-trihydroxy-4′-methoxyflavanone-3-*C*-glucose, 6-*C*-rhamnoside	— —	C_28_H_35_O_18_	[30]
**21**	5,7,3′-trihydroxy-4′-methoxyflavanone-3-*C*-rhamnose, 6-*C*-Arabinoside	— —	C_27_H_33_O_17_	[30]
**22**	5,7,4′-trihydroxyflavone-3-*C*-glucose, 6-*C*-rhamnoside	— —	C_27_H_33_O_17_	[30]
**23**	2″-*O*-α-l-rhamnoside-6-*C*-(6-*C*-Deoxy-ax-5-Methyl-xyl-hexan-4-carbonyl)-3′-methoxyluteolin	— —	C_27_H_28_O_14_	[17]
**24**	2″-*O*-α-l-rhamnoside-6-*C*-(3-Deoxyglucoside)-3′-methoxyluteolin	— —	C_28_H_32_O_14_	[22]
**25**	luteolin-6-*C*-glucoside	4261-42-1	C_21_H_20_O_11_	[17]
**26**	6,4′-dihydroxy-3′,5′-dimethoxyflavanone-7-*O*-glucoside	— —	C_24_H_26_O_11_	[31]
**27**	6,4′-dihydroxy-3′-methoxyflavanone-7-*O*-glucoside	— —	C_23_H_24_O_10_	[32]
**28**	isoorientin-2″-*O*-α-l-rhamnoglucoside	50980-94-4	C_27_H_30__O15_	[33]
**29**	5,7-dihydroxy-3′-methoxyflavanone-6-*C*-diglucoside	— —	C_28_H_32_O_15_	[34]
**30**	5-hydroxy-4′-methoxyflavanone-6-*C*-rhamnose-7-*O*-glucoside	— —	C_28_H_32_O_14_	[34]
**31**	chrysoeriol-6-*C*-β-boywinoside	— —	C_22_H_22_O_9_	[34]
**32**	homoeriodictyol-6-*C*-β-boivinose-7-*O*-β-glucopyranoside	— —	C_28_H_43_O_18_	[34]
**33**	homoeriodictyol-7-*O*-β-d-glucopyranoside	— —	C_22_H_22_O_11_	[34]
**34**	homoeriodictyol-6-*C*-β-fucoside	— —	C_22_H_22_O_10_	[35]
**35**	2″-*O*-α-l-rhamnose-6-*C*-(*trans*-5″-methyl-xyl-hexan-4-glucoside)-3′-methoxyluteolin	— —	C_28_H_30_O_14_	[22]
**36**	7,4′-dihydroxy-3′-methoxyflavanone-2″-*O*-α-l-rhamnose-6-*C*-fucoside	— —	C_27_H_30_O_15_	[36]
**37**	formononetin	485-72-3	C_16_H_12_O_4_	[37]
**38**	homoeriodictyol 7-*O*-glucoside	14982-11-7	C_22_H_24_O_11_	[34]
**39**	homoeriodictyol-6-*C*-β-boivinose-7-*O*-β-glucoside	— —	C_24_H_26_O_10_	[34]
**40**	homoeriodictyol-6-*C*-β-boivinoside	— —	C_22_H_22_O_9_	[34]
**41**	diosmetin	520-34-3	C_16_H_12_O_6_	[38]
**42**	schaftoside	51938-32-0	C_26_H_28_O_14_	[38]
**43**	robinin	301-19-9	C_33_H_40_O_19_	[39]
**44**	procyanidins	20347-71-1	C30H26O13	[39]
**45**	daidzein	486-66-8	C_15_H_10_O_4_	[39]
**46**	naringenin	480-41-1	C_15_H_12_O_5_	[39]
**47**	rutin	153-18-4	C_27_H_30_O_16_	[39]
**48**	quercetin	117-39-5	C_15_H_10_O_7_	[39]
**49**	catechin	154-23-4	C_15_H_14_O_6_	[39]
**50**	genistin	529-59-9	C_21_H_20_O_10_	[16]
**51**	prunetin 5-*O*-β-d-glucopyranoside	89595-66-4	C_22_H_22_O_10_	[16]
**52**	eriodictyol-7-*O*-glucoside	38965-51-4	C_21_H_22_O_11_	[16]
**53**	isovitexin 8-*C*-β-glucoside	23666-13-9	C_27_H_30_O_15_	[16]
**54**	apigenin-6,8-di-glucopyranoside	73140-47-3	C_25_H_26_O_13_	[16]
**55**	homoeriodictyol	69097-98-9	C_16_H_14_O_6_	[16]
**56**	violanthin	40581-17-7	C_27_H_30_O_14_	[16]
**57**	kaempferol-3-*O*-rhamnoside	83170-31-4	C_33_H_40_O_19_	[16]
**58**	8-*C*-(2-Rhamnosyl-6-deoxyhexopyranosulyl)-luteolin	933463-03-7	C_27_H_28_O_14_	[16]
**59**	isorhamnetin 3-*O*-neohesperidoside	55033-90-4	C_28_H_32_O_16_	[16]
**60**	quercetin 3,7-dimethyl ether-5-glucoside	44259668	C_23_H_24_O_12_	[16]
**61**	pectolinarigenin	520-12-7	C_17_H_14_O_6_	[16]
**62**	isorhamnetin 3,4′-diglucoside	5901757	C_28_H_32_O_17_	[16]
**63**	chrysin-7-*O*-β-d-glucuronide	35775-49-6	C_21_H_18_O_10_	[16]
**64**	astilbin	29838-67-3	C_21_H_22_O_11_	[16]
**65**	3′-deoxymaysin	44257705	C_27_H_28_O_13_	[16]
**66**	3,7-dihydroxy-3′,4′-dimethoxyflavone	5378832	C_17_H_14_O_6_	[16]
**67**	3′-deoxyderhamnosylmaysin	44257654	C_21_H_18_O_9_	[16]
**68**	quercetin-3,7,3′,4′-tetramethyl ether	1245-15-4	C_19_H_18_O_7_	[16]
**69**	kaempferol 3,7,4′-trimethyl ether	15486-34-7	C_18_H_16_O_6_	[16]
**70**	alternanthin	44258156	C_22_H_22_O_9_	[16]
**71**	engeletin	572-31-6	C_21_H_22_O_10_	[16]
**72**	cirsimaritin	6601-62-3	C_17_H_14_O_6_	[16]
**73**	cirsilineol	41365-32-6	C_18_H_16_O_7_	[16]
**74**	rhoifolin	17306-46-6	C_27_H3_0_O_14_	[16]
**75**	prunetrin	154-36-9	C_22_H_22_O_10_	[16]
**76**	2′-*O*-alpha-l-Rhamnosyl-6-*C*-quinovopyranosyl-luteolin	44257958	C_27_H_30_O_14_	[25]
**77**	2′-*O*-alpha-l-Rhamnosyl-6-*C*-fucosyl-luteolin	44257957	C_27_H_30_O_14_	[25]
**78**	derhamnosylmaysin	44257945	C_21_H_18_O_10_	[25]
**79**	3′-*O*-methylderhamnosylmaysin	44258171	C_22_H_20_O_10_	[25]
**80**	3′-Deoxymaysin	44257705	C_27_H_28_O_13_	[16]

**Table 2:** Chemical composition of sterols in corn silk.

No.	Serial Number	Name	CAS	Formula	Reference
1	**81**	stigmasterol	83-48-7	C_29_H_48_O	[42]
2	**82**	stigmastone	1058-61-3	C_29_H_48_O	[43]
3	**83**	sitostenone	1058-61-3	C_29_H_48_O	[43]
4	**84**	7α-Hydroxysitosterol	34427-61-7	C_29_H_50_O_2_	[34]
5	**85**	7β-Hydroxysitosterol	15140-59-7	C_29_H_50_O_2_	[34]
6	**86**	stigmast-5,22-3β,7α-diol	375649565	C_29_H_48_O_2_	[42]
7	**87**	β-sitosterol	83-46-5	C_29_H_50_O	[30]
8	**88**	stigmast-4-ene-3β,6β-diol	439985368	C_29_H_50_O_2_	[41]
9	**89**	ergosta-7,22-diene-3β,5α,6β-triol	12302764	C_28_H_46_O_3_	[34]
10	**90**	stigmast-4,22-diene-3β,6β-diol	167958-89-6	C_29_H_48_O_2_	[41]
11	**91**	stigmast-5-ene-3β,7α-diol	34427-61-7	C_29_H_50_O_2_	[41]
12	**92**	ergosterol endoperoxide	2061-64-5	C_28_H_44_O_3_	[41]
13	**93**	daucosterol-palmitate	542-44-9	C_19_H_38_O_4_	[34]
14	**94**	cholest-5-en-3-yl acetate	604-35-3	C_29_H_48_O_2_	[38]

**Table 3:** Chemical composition of terpenoids in corn silk.

No.	Serial Number	Name	CAS	Formula	Reference
1	**95**	costunolide	553-21-9	C_15_H_20_O_2_	[38]
2	**96**	friedelin	559-74-0	C_30_H_50_O_12_	[45]
3	**97**	α-amyrin	638-95-9	C_30_H_50_O_12_	[46]
4	**98**	α-terpineol	98-55-5	C_10_H_18_O	[47]
5	**99**	citronellol	106-22-9	C_10_H_20_O	[47]
6	**100**	6,11-oxidoacor-4-ene	— —	C_15_H_24_O	[47]
7	**101**	*trans*-pinocamphone	547-60-4	C_10_H_16_O	[47]
8	**102**	neo-iso-3-thujanol	— —	C_10_H_18_O	[47]
9	**103**	*cis*-sabinene hydrate	7712-82-5	C_10_H_18_O	[47]
10	**104**	pseudolaric acid E	— —	C_21_H_30_O_4_	[48]
11	**105**	19-hydroxy-*R*-kaurane-15-ene-17-carboxylic acid	— —	C_21_H_32_O_3_	[42]
12	**106**	17-hydroxy-*R*-kaurane-15-ene-19-oleic acid	— —	C_21_H_32_O_3_	[42]
13	**107**	3α-hydroxy-*R*-kaurane-15-ene-17-oleic acid-19-methyl carboxylate	— —	C_22_H_32_O5	[42]
14	**108**	ursolic acid	77-52-1	C_30_H_48_O_3_	[45]
15	**109**	3-*O*-Lauryl lactone	— —	C_22_H_32_O_4_	[49]
16	**110**	*R*-iosane-5β,15,16-triol	— —	C_20_H_36_O3	[42]
17	**111–118**	stigmaydene A–H	— —	— —	[48]
18	**119**	ent-16α,17-Dihydroxy-19-kauranoic acid	74365-74-5	C_20_H_32_O_4_	[48]
19–26	**120–124**	stigmaydene I–M	— —	— —	[50]
27	**125–128**	stigmane A–D	— —	— —	[51]
28–32	**129**	zeamalic acid A	— —	C_15_H_18_O_3_	[26]
33–36	**130**	zeamalic acid C	— —	C_15_H_18_O_3_	[26]
37	**131**	3-(4-hydroxyphenyl)-5,5-dimethyl-2-Cyclohexene-1-one	4045-07-2	C_24_H_37_N_3_O_2_	[51]
38	**132–136**	stigmene A–E	— —	— —	[50]
39	**137–140**	stigmene F–I	— —	— —	[50]
40–44	**141**	zealexin A3	134820458	C_15_H_21_O_3_	[50]
45–48	**142**	3-(4-hydroxyphenyl)-5,5-dimethyl-2-cyclohexen-1-one	— —	C_14_H_18_O_2_	[51]
49	**143**	β-carotene	7235-40-7	C_40_H_56_	[52]
50	**144**	zeaxanthin	144-68-3	C_40_H_56_O_2_	[53]

**Table 4:** Chemical composition of saponins in corn silk.

No.	Serial Number	Name	CAS	Formular	Reference
1	**145**	7α-hydroxy stigmast-3-*O*-β-d-glucopyranoside	112137-81-2	C_35_H_60_O_7_	[34]
2	**146**	stigmasterol-3-*O*-β-d-glucopyranoside	19716-26-8	C_35_H_58_O_6_	[45]
3	**147**	stigmast-3-*O*-β-d-glucopyranoside	474-58-8	C_35_H_60_O_6_	[45]

**Table 5:** Chemical composition of organic acids in corn silk.

No.	Serial Number	Name	CAS	Formula	Reference
1	**148**	phenylalanine	62056-68-2	C_9_H_11_NO_2_	[38]
2	**149**	d-tert-Leucine	26782-71-8	C_6_H_13_NO_2_	[54]
3	**150**	l-isoleucine	131598-62-4	C_6_H_13_NO_2_	[54]
4	**151**	l-aspartic acid	6899-03-2	C_4_H_7_NO_4_	[54]
5	**152**	dl-threonine	80-68-2	C_4_H_9_NO_3_	[54]
6	**153**	argininic acid	157-07-3	C_6_H_13_N_3_O_3_	[54]
7	**154**	proline	147-85-3	C_5_H_9_NO_2_	[54]
8	**155**	serine	302-84-1	C_3_H_7_NO_3_	[54]
9	**156**	valine	7004-03-7	C_5_H_11_NO_2_	[26]
10	**157**	l-lysine	56-87-1	C_6_H_14_N_2_O_2_	[54]
11	**158**	l-methionine	63-68-3	C_5_H_11_NO_2_S	[54]
12	**159**	l-glutamic acid	56-86-0	C_5_H_9_NO_4_	[54]
13	**160**	l-(−)-Tyrosine	55520-40-6	C_9_H_11_NO_3_	[54]
14	**161**	l-Histidine	71-00-1	C_6_H_9_N_3_O_2_	[54]
15	**162**	glycine-15N	7299-33-4	C_2_H_5_NO_2_	[54]
16	**163**	l-alanine	6898-94-8	C_3_H_7_NO_2_	[54]
17	**164**	chlorogenic acid	327-97-9	C_16_H_18_O_9_	[55]
18	**165**	oleic acid	112-80-1	C_18_H_34_O_2_	[56]
19	**166**	linoleic acid	60-33-3	C_18_H_32_O_2_	[57]
20	**167**	lactic acid	50-21-5	C_3_H_6_O_3_	[58]
21	**168**	docosanoic acid	112-85-6	C_22_H_44_O_2_	[58]
22	**169**	vanillic acid	121-34-6	C_8_H_8_O_4_	[58]
23	**170**	stearic acid	57-11-4	C_18_H_36_O_2_	[59]
24	**171**	palmitic acid-13C	287100-87-2	C_16_H_32_O_2_	[34]
25	**172**	*trans*-4-Hydroxycinnamic acid	4501-31-9	C_9_H_8_O_3_	[23]
26	**173**	formic acid	64-18-6	CH_2_O_2_	[58]
27	**174**	acetic acid	64-19-7	C_2_H_4_O_2_	[58]
28	**175**	succinic acid	110-15-6	C_4_H_6_O_4_	[58]
29	**176**	para-aminobenzoic acid	150-13-0	C_7_H_7_NO_2_	[60]
30	**177**	protocatechuic acid	99-50-3	C_7_H_6_O_4_	[60]
31	**178**	caffeic acid	501-16-6	C_9_H_8_O_4_	[61]
32	**179**	3-*O*-caffeoylquinic acid	1049703-62-9	C_16_H_18_O_9_	[61]
33	**180**	ferulic acid	1135-24-6	C_10_H_10_O_4_	[61]
34	**181**	quinic acid	77-95-2	C_7_H_12_O_6_	[61]
35	**182**	citric acid	77-92-9	C_6_H_8_O_7_	[61]
36	**183**	6-Hydroxypurine	146469-94-5	C_5_H_4_N_4_O	[61]
37	**184**	uridine	58-96-8	C_9_H_12_N_2_O_6_	[61]
38	**185**	galloylglucose	13186-19-1	C_13_H_16_O_10_	[61]
39	**186**	guanosine	85-30-3	C_10_H_13_N_5_O_5_	[61]
40	**187**	2-deoxy-d-guanosine monohydrate	312-693-72-4	C_10_H_13_N_5_O_4_	[61]
41	**188**	γ-GLU-PHE	7432-24-8	C_14_H_18_N_2_O_5_	[61]
42	**189**	l(−)-tryptophan	73-22-3	C_11_H_12_N_2_O_2_	[61]
43	**190**	5-hydroxyiosphthalic acid	618-83-7	C_8_H_6_O_5_	[61]
44	**191**	2-(β-d-Glucopyranosyloxy)-3-(4-hydroxyphenyl)propanoic acid	9602775	C_15_H_20_O_9_	[61]
45	**192**	2-(*E*)-*O*-feruloyl-d-galactaric acid	14104340	C_16_H_18_O_11_	[61]
46	**193**	5-(Isopropoxymethyl)-2-furoic acid	3926408	C_9_H_12_O_4_	[61]
47	**194**	dicaffeoyltartaric acid	70831-56-0	C_22_H_18_O_12_	[61]
48	**195**	2-caffeoylcitric acid	5280552	C_15_H_14_O_10_	[61]
49	**196**	8-methoxy-2,3-dihydro-1,4-benzodioxine-6-carboxylic acid	4962316	C_10_H_10_O_5_	[61]
50	**197**	4-(2-Hydroxyethyl)benzoic acid	46112-46-3	C_9_H_10_O_3_	[61]
51	**198**	2-furanacrylic acid	539-47-9	C_7_H_6_O_3_	[61]
52	**199**	12-oxo-PDA	5280411	C_18_H_28_O_3_	[61]
53	**200**	12-HETE	71030-37-0	C_20_H_32_O_3_	[25]
54	**201**	(−)-jasmonic acid	6894-38-8	C_12_H_18_O_3_	[25]
55	**202**	(s)-(+)-abscisic acid	21293-29-8	C_15_H_20_O_4_	[25]

**Table 6:** Chemical composition of polysaccharides and other ingredients in corn silk.

No.	Serial Number	Name	CAS	Formula	Reference
1	**203**	allantoin	97-59-6	C_4_H_6_N_4_O_3_	[63]
2	**204**	vanillin	121-33-5	C_8_H_8_O_3_	[63]
3	**205**	6,10,14-Trimethyl-5,9,13-pentadecatrien-2-one	762-29-8	C_18_H_30_O	[38]
4	**206**	6-methoxybenzo[d]isoxazole-3-carboxylic acid	28691-48-7	C_9_H_7_NO_4_	[58]
5	**207**	adenosine	58-61-7	C_10_H_13_N_5_O_4_	[58]
6	**208**	guanine	73-40-5	C_5_H_5_N_5_O	[58]
7	**209**	uracil	66-22-8	C_4_H_4_N_2_O_2_	[58]
8	**210**	dextrose	492-62-6	C_6_H_12_O_6_	[42]
9	**211**	l-Rhamnose	6155-35-7	C_6_H_14_O_6_	[23]
10	**212**	d-mannopyranose	530-26-7	C_6_H_12_O_6_	[23]
11	**213**	d-Galactose	59-23-4	C_6_H_12_O_6_	[23]
12	**214**	dl-Xylose	25990-60-7	C_5_H_10_O_5_	[23]
13	**215**	d-(+)-Fucose	3615-37-0	C_6_H_12_O_5_	[23]
14	**216**	d-Ribose	50-69-1	C_5_H_10_O_5_	[23]
15	**217**	l-(+)-Ribose	24259-59-4	C_5_H_10_O_5_	[23]
16	**218**	bovolide	774-64-1	C_11_H_16_O_2_	[64]
17	**219**	5,6-Dihydro-1*H*-imidazo [4,5-d]pyridazine-4,7-dione	6293-09-0	C_5_H_4_N_4_O_2_	[61]
18	**220**	(2*S*)-2-(β-d-Glucopyranosyloxy)-3-hydroxypropyl butyrate	9089566	C_13_H_24_O_9_	[61]
19	**221**	5,6-Bis[(2,4-dinitrophenyl)hydrazono]-1,2,3,4-hexanetetrol	54027-04-2	C_18_H_18_N_8_O_12_	[61]
20	**222**	3,4-dihydroxybenzaldehyde	134998-43-9	C_7_H_6_O_3_	[61]
21	**223**	neochlorogenic acid	906-33-2	C_16_H_18_O_9_	[61]
22	**224**	cryptochlorogenic acid	905-99-7	C_16_H_18_O_9_	[61]
23	**225**	Evolvoid A	16723783	C_19_H_28_O_10_	[61]
24	**226**	2,2-Dimethyl-3-phenylpentanedioic acid hydrate (1:1)	2029423	C_13_H_18_O_5_	[61]
25	**227**	guaifenesin	93-14-1	C_10_H_14_O_4_	[61]
26	**228**	1-*O*-*p*-coumaroylglycerol	106055-11-2	C_12_H_14_O_5_	[61]
27	**229**	feruloylisocitric acid	129661569	C_16_H_16_O_10_	[61]
28	**230**	2-(2-((4-(2-Hydroxyethoxy)-2-butynyl)oxy)ethoxy)ethanol	84282-21-3	C_10_H_18_O_5_	[61]
29	**231**	{(3*R*,5*R*)-5-[(1*S*)-1-Hydroxypropyl]tetrahydro-3-furanyl}acetic acid	9681387	C_9_H_16_O_4_	[61]
30	**232**	(2*E*)-3-(7-Propoxy-3,4-dihydro-2*H*-chromen-3-yl)acrylic acid	10826028	C_15_H_18_O_4_	[61]
31	**233**	4-[(4-tert-butylcyclohexyl)oxy]-4-oxobutanoic acid	148114-19-6	C_14_H_24_O_4_	[61]
32	**234**	2-Isopropyl-5-methylhexanedioic acid	39668-86-5	C_11_H_20_O_2_	[61]
33	**235**	3,4-Dihydroxy-2-isopropyl-5-methylcyclohexanecarboxylic acid	28288176	C_11_H_20_O_4_	[61]
34	**236**	(+)-Aspicilin	52461-05-9	C_18_H_32_O_5_	[61]
35	**237**	(2*R*)-2-Hydroxy-4-[(1*S*,4*R*,6*R*)-4-hydroxy-2,2,6-trimethylcyclohexyl]butanoic acid	16216665	C_13_H_24_O_4_	[61]
36	**238**	(8*E*,12*Z*)-10,11-Dihydroxy-8,12-octadecadienoic acid	27025513	C_18_H_32_O_4_	[61]
37	**239**	cespitularin Q	101408387	C_20_H_30_O_4_	[25]
38	**240**	(+)-Gingerol	1391-73-7	C_17_H_26_O_4_	[61]
39	**241**	seimatopolide B	57409556	C_18_H_3_O_4_	[61]
40	**242**	pleocarpenone	102158596	C_14_H_24_O_3_	[61]
41	**243**	13-Hydroxy-13-(hydroxymethyl)podocarpan-3-one	28284391	C_18_H_30_O_3_	[61]
42	**244**	indole-3-acetic acid	87-51-4	C_10_H_9_NO_2_	[61]
43	**245**	6-Methoxybenzoxazolin-2(3*H*)-one	10772	C_8_H_7_NO_3_	[61]
44	**246**	*trans*-Zeatin	1637-39-4	C_10_H_13_N_5_O	[61]
45	**247**	alloimperatorin	642-05-7	C_16_H_14_O_4_	[61]
46	**248**	subaphyllin	501-13-3	C_14_H_20_N_2_O_3_	[61]
47	**249**	lumichrome	1086-80-2	C_12_H_10_N_4_O_2_	[61]
48	**250**	gibberellin A17	5460657	C_20_H_26_O_7_	[61]
49	**251**	OPC-4:0	5716900	C_14_H_22_O_3_	[61]
50	**252**	13(*S*)-Hydroperoxylinolenic acid	5497123	C_18_H_30_O_4_	[61]
51	**253**	gibberellin A9	427-77-0	C_19_H_24_O_4_	[61]
52	**254**	gibberellin A3	77-06-5	C_19_H_22_O_6_	[61]
53	**255**	gibberellin A8	7044-72-6	C_19_H_24_O_7_	[61]
54	**256**	β-Tocotrienol	490-23-3	C_28_H_42_O_2_	[61]
55	**257**	gibberellin A24	19427-32-8	C_20_H_26_O_5_	[61]
56	**258**	gibberellin A1	545-97-1	C_19_H_24_O_6_	[61]
57	**259**	gibberellin A14	429678-85-3	C_20_H_28_O_5_	[61]
58	**260**	gibberellin A12	1164-45-0	C_20_H_28_O_4_	[61]
59	**261**	3-Oxo-2-(2-entenyl)cyclopentaneoctanoic acid	5280729	C_18_H_30_O_3_	[61]
60	**262**	rhamnosyl urea	— —	C13H24N2O9	[27]
61	**263**	1,3-dirhamnosyl urea	— —	C8H16N2O5	[27]
62	**264**	l-mannosehydrat	10030-85-0	C6H14O6	[34]
63	**265**	caffeoyl glucoside	5281761	C_15_H_18_O_9_	[61]
64	**266**	salicylic acid	69-72-7	C_7_H_6_O_3_	[61]
65	**267**	vanillic aldehyde	8014-42-4	C_8_H_8_O_3_	[61]
66	**268**	(*E*)-*p*-coumaric acid	501-98-4	C_9_H_8_O_3_	[61]
67	**269**	alnusone	52330-11-7	C_19_H_18_O_3_	[45]
68	**270**	eugenol	97-53-0	C_10_H_12_O_2_	[47]
69	**271**	(2*S*,3*R*,4*R*,5*E*)-5-[(2*E*)-{6-Amino-9-[(2*R*,3*R*,4*S*,5*R*)-3,4-dihydroxy-5-(hydroxymethyl)tetrahydro-2-furanyl]-1,9-dihydro-2*H*-purin-2-ylidene}hydrazono]-1,2,3,4-pentanetetrol	— —	C_15_H_23_N_7_O_8_	[61]
70	**272**	1-(4-Amino-1,2,5-oxadiazol-3-yl)-5-(methoxymethyl)-*N*’-(2-oxo-2*H* indol-3-yl)-1*H*-1,2,3-triazole-4-carbohydrazide	— —	C_15_H_13_N_9_O_4_	[61]
71	**273**	ylamide	— —	C_18_H_24_N_4_O_11_	[61]
72	**274**	6-*O*-(2-Hydroxyhexanoyl)-d-glucopyranose	— —	C_12_H_22_O_8_	[61]
73	**275**	dimethyl3,3-(2,5-dihydroxy-3,6-dioxo-1,4-cyclohexadiene-1,4-diyl)bis [3-(3-hydroxy-4-methoxyphenyl)propanoate]	— —	C_28_H_28_O_12_	[61]
74	**276**	5-Hydroxy-2-(4-hydroxy-3-methoxyphenyl)-4-oxo-4H-chromen-7-yl2-*O*-β-d-threo-hexopyranuronosyl-β-d-threo hexopyranosiduronic acid	— —	C_28_H_28_O_18_	[61]
75	**277**	4-[Bis(2-hydroxy-4-oxo-4*H*-chromen-3-yl)methyl]phenyl(2*E*)-3-(3,4-dihydroxyphenyl)acrylate	— —	C_34_H_22_O_10_	[61]
76	**278**	(1*S*,3*R*,4*R*,5*R*)-1-{[(3*S*)-3-(3,4-Dihydroxyphenyl)-3-methoxypropanoyl]oxy}-3-{[(2*E*)-3-(3,4-dihydroxyphenyl)-2-propenoyl]oxy}-4,5-dihydroxycyclohexanecarboxylic acid	— —	C_26_H_28_O_13_	[61]
77	**279**	(1*S*,3*R*,4*R*,5*R*)-3-{[(3*S*)-3-(3,4-Dihydroxyphenyl)-3-methoxypropanoyl]oxy}-1-{[(2*E*)-3-(3,4-dihydroxyphenyl)-2-propenoyl]oxy}-4,5-dihydroxycyclohexanecarboxylic acid	— —	C_26_H_28_O_13_	[61]
78	**280**	5-Hydroxy-2-(4-methoxyphenyl)-4-oxo-4*H*-chromen-7-yl 2-*O*-(6-deoxy-α-l-mannopyranosyl)-β-d-glucopyranosiduronic acid	— —	C_28_H_30_O_15_	[61]
79	**281**	(3*R*,4*R*,5*E*,9*S*,10*R*)-9-Hydroxy-3-methyl-2-oxo-10-pentyl-3,4,7,8,9,10-hexahydro-2*H*-oxecin-4-yl acetate	— —	C_17_H_28_O_5_	[61]
80	**282**	(4*S*,6*S*,7*Z*,9*S*,10*S*)-4,6,9-Trihydroxy-10-nonyl-3,4,5,6,9,10-hexahydro-2*H*-oxecin-2-one	— —	C_18_H_32_O_5_	[61]
81	**283**	(3*E*,5*S*,6*R*,7*S*,18*S*)-5,6,7-Trihydroxy-18-methyloxacyclooctadec-3-en-2-one	— —	C_18_H_32_O_5_	[61]
82	**284**	5-{(2*R*,5*R*)-5-[(1*R*)-1-Hydroxynonyl]tetrahydro-2-furanyl}pentanoic acid	— —	C_18_H_34_O_4_	[25]

**Table 7:** The pharmacological actions and related mechanisms of corn silk.

No.	Pharmacological Actions	Mechanisms	References
1	Hyperglycemic effect	Callback sugar metabolism, fat metabolism, amino acid metabolic pathways of chenodeoxycholic acid, 5-HIAA, (*R*)-3-hydroxybutyric acid, argininosuccinic acid, 4,6-dihydroxyquinoline, LTB4, and other sites, improve glucose, lipid, and amino acid metabolism disorders	[95]
		Repair the pathological changes in the liver, kidney, and pancreas	[67]
		Exhibits good hypoglycemic effect on type II diabetic mice, and has a good inhibitory effect on α-glucosidase and α-amylase activities	[68]
2	Antigout action	Inhibit the expression of IL-1β and decrease serum uric acid level	[71]
		Reduce the production of UA, BUN, and Cr by reducing the concentration of Xanthine oxidase (XOD) and PRPS	[73]
3	Liver protection	Down-regulation of Smad3 mRNA expression in liver tissue reduces the secretion of ECM and inhibits the development of liver fibrosis	[74]
		Reduce MDA content in serum and liver and increase SOD activity, the mechanism may be related to anti-lipid peroxidation	[75]
4	Anti-hyperlipidemia	Increase LPL and HL enzyme activities, reduce TC, TG, and LDL-C contents, and increase HDL-C content to regulate blood lipid balance and increase SOD, GSH-px, and CAT anti-oxidant enzyme activities	[78]
5	Anti-oxidant	Increase anti-oxidant enzyme levels and inhibit lipid peroxidation	[96]
		Against oxidative stress through the upregulation of Nrf2	[97]
6	Anti-inflammatory	Enhance T-cell-mediated immune response and decrease inflammatory factors	[47]
		Reduce the expression of TNF-α and IL-1β	[88]
7	Kidney protection	Decrease UA production by interfering with XOD	[47]
		PI3K/AKT and NF-κB signaling were the pivotal pathways	[98]
8	Antihypertensive	Vascular expansion in low-concentration	[90]
9	Anti-cancer	Anti-cancer through the serine/threonine kinases (Akt)/lipid kinases (PI3Ks) pathway	[99]
10	Protecting the reproductive system	Recover the amounts of sex hormones and sperm count to normal conditions by reducing lipid peroxidation	[93]
